# Supplementary material for: In their own words: A qualitative examination of student experiences with high-impact practices during the second-year transition
Source: PLoS One. 2026 Jan 16;21(1):e0340395. doi: 10.1371/journal.pone.0340395 (PMC12810777; doi:10.1371/journal.pone.0340395)
Supplement: Attachment — Submitted filename: Review result.pdf [file pone.0340395.s001.pdf]

“In their own words: A qualitative examination of undergraduate student experiences with high-impact practices during the second-year transition”

The manuscript presents a qualitative analysis of second-year undergraduate students' experiences in a summer-bridge program that incorporated high-impact practices. The authors claim that structured support through academic, social, professional, and personal engagement significantly contributes to student development and potentially mitigates the “sophomore slump.” This is a valuable and timely contribution to higher education research, especially given the limited focus on second-year student transitions in existing literature. The study is significant for informing future program design and institutional policies.
The claims are appropriately situated within the existing literature. The manuscript includes a comprehensive and up-to-date literature review that highlights the gap in research related to second-year students. The authors fairly represent both the strengths and limitations of prior work, especially in terms of its emphasis on first-year interventions. The references are current and diverse, demonstrating an awareness of both theoretical and applied studies in the field.

The data and analysis sufficiently support the claims made in the paper. The authors used a phenomenographic approach with clear justification, systematically coded student reflections, and ensured analytic rigor through multiple raters and inter-rater reliability metrics. While the nature of the qualitative data limits generalizability, the authors provide rich evidence in the form of student quotations and detailed analysis that align with their conclusions. Claims are appropriately cautious and do not overstate the findings.

The methods are clearly explained, particularly the data collection and coding processes. While this is not a clinical trial or quantitative experimental study, the coding protocol used for qualitative analysis might benefit the research community if provided as supplementary material or in a data repository. No deviations from established protocols are present since this is an exploratory qualitative study.

The manuscript is suitable for publication pending minor revisions. It demonstrates strong potential and relevance to the field. The main revision needed is related to the data availability policy, which currently does not meet PLOS ONE requirements. No original data have been deposited in a public repository. The authors state that data are available upon reasonable request, which does not comply with PLOS ONE's data sharing policy. Anonymized reflection samples or the full dataset (if possible) should be uploaded to a repository, or the authors must provide a valid ethical/legal justification for withholding public access. However, the ethical protocol and IRB exemption are stated and consistent with institutional and federal guidelines. No deviations from ethical standards were noted.

Lastly, the methodology is described with sufficient detail for replication in other institutional contexts. The authors clearly outline how reflections were collected, how

coding was conducted, and how inter-rater reliability was established. The manuscript is well organized, clearly written, and accessible to a broad audience, including non-specialists in qualitative research or education policy. Terminology is appropriately explained, and the flow of the manuscript is logical and easy to follow.
