# Supplementary material for: In their own words: A qualitative examination of student experiences with high-impact practices during the second-year transition
Source: PLoS One. 2026 Jan 16;21(1):e0340395. doi: 10.1371/journal.pone.0340395 (PMC12810777; doi:10.1371/journal.pone.0340395)
Supplement: Attachment — Submitted filename: PONE-D-24-02966R1 Responses.docx [file pone.0340395.s003.docx]

August 21, 2025

Dear Dr. El-Sayed,

We appreciate the thorough comments and the helpful suggestions for strengthening this manuscript (PONE-D-24-02966). Because of the extensive comments, we have organized them to streamline the revision process. Our responses are included with the label [Response] and in blue text below. Editor and reviewer comments have been labeled should they wish to revisit their particular comments or see how their comments are related to those from other reviewers.

**Editorial Comments**

Please ensure that your manuscript meets PLOS ONE's style requirements, including those for file naming.

**[Response]:** Thank you for these resources. We have updated the manuscript to align with PLOS ONE’s style requirements based on the resources provided.

Please provide additional details regarding participant consent. In the ethics statement in the Methods and online submission information, please ensure that you have specified what type you obtained (for instance, written or verbal, and if verbal, how it was documented and witnessed). If your study included minors, state whether you obtained consent from parents or guardians. If the need for consent was waived by the ethics committee, please include this information.

**[Response]:** Thank you for this comment. The paragraph on Line 288 specifies that participant consent was not required. The Institutional Review Board (IRB) under the Human Research Protection Program (HRPP) at the University of California San Diego (UCSD) determined that the project met the criteria for Not Human Subjects Research (NHSR). As such, the study was not considered human subjects research, and the requirement for obtaining participant consent was waived.

Thank you for stating the following financial disclosure:
“The summer-bridge program in this study was supported by the University of California San Diego.”

Please state what role the funders took in the study. If the funders had no role, please state: "The funders had no role in study design, data collection and analysis, decision to publish, or preparation of the manuscript."

**[Response]:** Thank you for pointing this out. We added this statement in our financial disclosure, as the funders played no role in the study.

Thank you for stating the following in the Acknowledgments Section of your manuscript:
“We thank the students from the summer-bridge program for sharing their perspectives and experiences. The summer-bridge program was supported by University of California San Diego.”

We note that you have provided funding information that is not currently declared in your Funding Statement. However, funding information should not appear in the Acknowledgments section or other areas of your manuscript. We will only publish funding information present in the Funding Statement section of the online submission form.
Please remove any funding-related text from the manuscript and let us know how you would like to update your Funding Statement. Currently, your Funding Statement reads as follows:
“The summer-bridge program in this study was supported by the University of California San Diego.”

**[Response]:** Thank you for this clarification. We have removed the mention of funding from the acknowledgments section and removed the Funding Statement from the manuscript.

The funding statement should read as follows: The summer-bridge program used as a study site in this paper was financially supported by the University of California San Diego. The funders had no role in study design, data collection and analysis, decision to publish, or preparation of the manuscript.

In the online submission form, you indicated that “The datasets created and/or analyzed for this study are available from the corresponding author upon reasonable request.”

All PLOS journals now require all data underlying the findings described in their manuscript to be freely available to other researchers, either 1. In a public repository, 2. Within the manuscript itself, or 3. Uploaded as supplementary information.
This policy applies to all data except where public deposition would breach compliance with the protocol approved by your research ethics board. If your data cannot be made publicly available for ethical or legal reasons (e.g., public availability would compromise patient privacy), please explain your reasons on resubmission and your exemption request will be escalated for approval.

**[Response]:** Thank you for the clarifying the open access data policy of the journal. We have created a repository of all raw student reflections (with identified information removed) using the platform Open Science Framework platform.

The link to the repository that can be accessed by readers is here: <https://osf.io/8p3yu/?view_only=c2252a84b94e47a2944adeb306dc11ae>

Please review your reference list to ensure that it is complete and correct. If you have cited papers that have been retracted, please include the rationale for doing so in the manuscript text, or remove these references and replace them with relevant current references. Any changes to the reference list should be mentioned in the rebuttal letter that accompanies your revised manuscript. If you need to cite a retracted article, indicate the article’s retracted status in the References list and also include a citation and full reference for the retraction notice.

**[Response]:** We have not retracted any references from the original submission. However, we have added the following new literature sources at the request of Reviewers 1 and 4. These have been added as in-text citations and in the References list.

McDaniel A, Halper LR, Van Jura M, Fines B, Whittington MS. Increasing Student Retention Rates Through Engagement in a Comprehensive Second-Year Program. J Coll Stud Retent Res Theory Pract. 2024;26(2):401–21.

Young DG, Schreiner LA, McIntosh EJ. Investigating Sophomore Student Success: National Survey of Sophomore-Year Initiatives and the Sophomore Experiences Survey, 2014. Columbia, SC: University of South Carolina, National Resource Center for The First-Year Experience & Students in Transition; 2015

Price-Williams S, Sasso P. College Sophomore Students’ Self-Efficacy and Intent to Persist. J Coll Orientat Transit Retent. 2024. 31(2).

Price-Williams S, Sasso PA. A Cross-Case Analysis of Sophomore Students’ Reflections on Self- Efficacy: Signals for Innovative Sophomore Programming. High Educ Polit Econ. 2025;11(2).

Perez M. An Examination of University Sophomore Students’ Thriving Factors, Second-Year Experiences, and Student Success Outcomes. Res Issues Contemp Educ. 2020;5(1):1–24.

Kranzow J, Foote SM. Engaging Sophomores Through Curricular and Cocurricular Initiatives. New Dir High Educ. 2018; 183:71–83.

The manuscript presents a qualitative analysis of second-year undergraduate students'

experiences in a summer-bridge program that incorporated high-impact practices. The

authors claim that structured support through academic, social, professional, and personal

engagement significantly contributes to student development and potentially mitigates the

“sophomore slump.” This is a valuable and timely contribution to higher education

research, especially given the limited focus on second-year student transitions in existing

literature. The study is significant for informing future program design and institutional

policies.

The claims are appropriately situated within the existing literature. The manuscript

includes a comprehensive and up-to-date literature review that highlights the gap in

research related to second-year students. The authors fairly represent both the strengths

and limitations of prior work, especially in terms of its emphasis on first-year interventions.

The references are current and diverse, demonstrating an awareness of both theoretical

and applied studies in the field.

The data and analysis sufficiently support the claims made in the paper. The authors used

a phenomenographic approach with clear justification, systematically coded student

reflections, and ensured analytic rigor through multiple raters and inter-rater reliability

metrics. While the nature of the qualitative data limits generalizability, the authors provide

rich evidence in the form of student quotations and detailed analysis that align with their

conclusions. Claims are appropriately cautious and do not overstate the findings.

The methods are clearly explained, particularly the data collection and coding processes.

While this is not a clinical trial or quantitative experimental study, the coding protocol used for qualitative analysis might benefit the research community if provided as supplementary material or in a data repository. No deviations from established protocols are present since this is an exploratory qualitative study.

Lastly, the methodology is described with sufficient detail for replication in other

institutional contexts. The authors clearly outline how reflections were collected, how

coding was conducted, and how inter-rater reliability was established. The manuscript is well organized, clearly written, and accessible to a broad audience, including non-specialists in qualitative research or education policy. Terminology is appropriately explained, and the flow of the manuscript is logical and easy to follow.

**[Response]:** Thank you for this overall positive assessment of our manuscript!

The main revision needed is related to the data availability policy, which currently does not meet PLOS ONE requirements. No original data have been deposited in a public repository. The authors state that data are available upon reasonable request, which does not comply with PLOS ONE's data sharing policy. Anonymized reflection samples or the full dataset (if possible) should be uploaded to a repository, or the authors must provide a valid ethical/legal justification for withholding public access. However, the ethical protocol and IRB exemption are stated and consistent with institutional and federal guidelines. No deviations from ethical standards were noted.

**[Response]:** Thank you again for clarifying the journal’s data availability policy. We have deposited anonymized reflection data in a public repository, which can be found here: <https://osf.io/8p3yu/?view_only=c2252a84b94e47a2944adeb306dc11ae>

**Reviewer 1**

The article under consideration for publication is well-structured, addresses an interesting study problem, and makes a significant contribution to the design of educational practices relevant to the development of higher education students, particularly those in transition to the second year. The qualitative methodological approach employed by the authors, which involves giving students a voice, enables a more integrated or holistic understanding of how students perceive these practices and whether they are meaningful to them.

The title of the article is an accurate reflection of its content.

In the introduction, the authors present a detailed account of the background to the study and the research question, providing a comprehensive overview of the context in which it was conducted and its significance in terms of educational practice and policy. This framework enables the reader to comprehend the scientific and social significance of the study.

In the methodological section, the authors provide a comprehensive account of the processes and procedures employed to develop the study, delineate the methodological perspective adopted (phenomenology) and justify its selection, identify the method of data collection in detail, and furnish a detailed description of how the participants were recruited, including information on time span (dates) and procedures, with particular attention to those aimed at guaranteeing reliability in the analysis.

With regard to data analysis, the present study describes each step of the content analysis of the students' reflections, as well as the process by which the conclusions were generated. This includes the criteria used to generate the themes, the number of coders involved, and the means of ensuring consistency between evaluators. The manuscript addresses ethical issues in an appropriate manner.

The presentation of the results adheres to the themes that emerged from the analysis. It offers a reflective analysis of the data obtained, augmented with quotations from the participants that substantiate the themes and the authors' interpretations.

**[Response]:** Thank you for this overall very positive assessment of the manuscript!

The literature review addresses the existing research on the problem and identifies the strengths and limitations of other studies in their approach to the topic. Despite the dearth of studies involving higher education students during the second-year transition, it would have been beneficial to incorporate some more recent literature into the analysis.

It is recommended that the Literature Review Section include references from more recent publications. Furthermore, the references included in the references list/section should be reviewed and corrected.

**[Response]:** Thank you for this suggestion. We have conducted additional literature review to identify additional recent sources that support our claims, as listed in the response to the editor’s comments above. These have been added as in-text citations and in the references.

**Reviewer 2**

This is a well-executed and timely study that adds meaningful qualitative insights into second-year undergraduate experiences, a population often overlooked in higher education research. The use of a phenomenographic framework is appropriate and thoughtfully applied. The authors do an excellent job of connecting their findings to existing literature and highlighting practical implications for institutional policy and student support.

**[Response]:** Thank you for this positive assessment!

However, to comply with PLOS ONE's data policy, the authors should ensure that anonymized reflection data or a suitable sample of coded data is made openly accessible through a repository or supplementary material or clearly justify any restrictions on sharing.

**[Response]:** Thank you for your comment regarding compliance with PLOS ONE's data policy. We appreciate the importance of data transparency and reproducibility. We have now included a de-identified repository of the reflection data. We hope this satisfies the journal’s data availability requirements while respecting participant privacy.

Although the manuscript does not include statistical analyses (as it is not required for the methodology） because this study was Qualitative, however, this should be clearly stated in the limitations to avoid confusion for readers expecting like quantitative measures.

**[Response]:** Thank you for this thoughtful suggestion. We recognize the importance of acknowledging the scope and boundaries of our methodological choices. However, we have chosen not to frame the absence of statistical analysis as a limitation, as doing so may unintentionally suggest that quantitative approaches are inherently preferable. This could risk misrepresenting the epistemological grounding of our qualitative design. To provide greater clarity for readers, we have added a sentence on line 109 in the final paragraph of the Introduction to explain our rationale for selecting a qualitative approach.

**Reviewer 3**

However, it would be useful for readers to know whether some of the students had the opportunity to use research skills as part of the project because you mentioned R1 doctoral university as part of the data collection site.

**[Response]:** Thank you for this question. We have added a sentence in the Methods on Line 198 clarifying that while an R1 institution was a data collection site, undergraduate research training was not a focus of this program. A few students may have been part of research laboratories and reflected on the experience in their professional development reflections, but these experiences were not systematically captured or emphasized within the scope of the program or our data collection.

Phenomenography requires the use of diagrams, tables or figures. Your readers will find a diagrammatic representation of your results helpful in the results section.

**[Response]:** Thank you for this excellent suggestion. We agree that a visual representation can enhance readers' understanding of the phenomenographic outcomes. In response, we have added a diagram summarizing the phenemographic approach (now Fig 1) and a diagram of our application of Variation Theory in this current study (now Fig 2). We hope these additions provide a clearer and more accessible overview of our findings.

More information is required on the mandatory weekly reflections from the participants. You selected students based on their academic performance. It is interesting to note that ethical clearance deemed this project a quality improvement.

**[Response]:** Thank you for your comment. The full reflection prompt template is provided in Figure 3. We have also added a brief clarification on Line 208 that students were selected based on pace of the academic progress (i.e., enrolling in fewer than the requisite number of credits per term to be on track for a four-year graduation), rather than on academic performance (e.g. course grades).

**Reviewer 4**

The frequent use of a few specific sources (e.g. [31, 62]) indicates an excessive reliance on a limited number of studies. More diversity in citations could have strengthened the arguments.

**[Response]:** Thank you for this comment, which aligns with similar feedback from Reviewer 1. We have reviewed the manuscript and expanded the literature base to include a broader range of sources that support and contextualize our arguments.

The abstract does not fully identify key findings, such as the superiority of academic, personal, and social outcomes over professional outcomes. Also, there is no mention of the concept of “sophomore slump,” which is prominent in the main text.

**[Response]:** Thank you for this helpful comment. We have revised the abstract to allude to the “sophomore slump” phenomenon described in the Literature Review and more clearly highlight the key findings on the less salient professional outcomes.

The article is overly long at 8,352 words (including references), especially for a qualitative study. The literature review and discussion sections could be shortened to improve readability and focus.

Some material in the literature review, findings, and discussion sections is repetitive. For example, descriptions of the four dimensions of student development (academic, social, professional, and personal) are overly repeated across sections.

**[Response]:** Thank you for this feedback. We have reviewed the manuscript and shortened relevant parts of the literature review, results, and discussion section to reduce redundancy and improve focus.

Limitations of the study sample:
The study was conducted at only one university, and its sample (133 students) may not be representative of more diverse populations at other institutions. This limitation reduces the generalizability of the findings.
Lack of long-term outcomes:
The article did not examine long-term outcomes, such as graduation rates or student retention. This limits the scientific value of the study for evaluating the effectiveness of the program.

**[Response]:** We appreciate the reviewer's attention to these important limitations. These limitations are explicitly discussed in our manuscript. Given that this is a foundational qualitative study exploring student perceptions of high-impact practices, we believe our findings provide valuable insights for program development while appropriately acknowledging these constraints for future research.

Inadequate explanation of consent: Although it is noted that consent from participants was not required due to the lack of identifying information, more explanation could have been provided about the ethical considerations associated with using students’ personal reflections.

**[Response]:** Thank you for this suggestion. We have added clarification in the manuscript regarding the ethical considerations involved in using students’ personal reflections, including the removal of identifying information and restricted access to the data (Line 334).

Limited results in the professional dimension:
The findings related to professional development are less prominent than other dimensions (academic, social, personal). The article could have analyzed this weakness in more depth or explained its possible reasons.

**[Response]:** Thank you for this helpful observation. We have added further explanation in the paragraph on Line 601 to address the limited findings in the professional development dimension, including potential reasons related to timing of professional experiences.

The suggestion for longitudinal studies in the discussion section is general and does not provide specific details about the methods or expected results.

**[Response]:** Thank you for this helpful comment, we have added additional details regarding potential approaches to these longitudinal studies beginning on Line 624.

Some technical terms, such as "variation theory" or "phenomenography," may not be entirely clear to non-specialist readers because sufficient explanations are not provided in the text.

**[Response]:** Thank you for pointing this out. We have revised the manuscript to include brief yet accessible explanations of “phenomenography” and “variation theory” to provide more clarity for non-technical readers, as well as include citations to the seminal conceptualization of phenomenography put forth by Marton.

Marton F. Phenomenography — Describing conceptions of the world around us. Instr Sci. 1981;10(2):177–200.

Marton F. A research approach to investigating different understandings of reality. J Thought. 1986;21(3):28–49

Sincerely,

Stanley M. Lo, Ph.D.*

Teaching Professor

Cell and Developmental Biology

Mathematics and Science Education

University of California San Diego

Corresponding author: [smlo@ucsd.edu](mailto:smlo@ucsd.edu)
