# Supplementary material for: In their own words: A qualitative examination of student experiences with high-impact practices during the second-year transition
Source: PLoS One. 2026 Jan 16;21(1):e0340395. doi: 10.1371/journal.pone.0340395 (PMC12810777; doi:10.1371/journal.pone.0340395)
Supplement: Attachment — Submitted filename: PONE-D-24-02966R2 Responses.docx [file pone.0340395.s004.docx]

December 10, 2025

Dear Dr. El-Sayed,

We appreciate the additional comments for strengthening this manuscript (PONE-D-24-02966R1). Our responses are included with the label [Response] and in blue text below.

**Reviewer 1**

All proposed amendments have been integrated into the manuscript, resulting in a revised and enhanced version. With these modifications in place, I believe the article is now ready for publication.

**Reviewer 3**

Thank you for taking the time and effort to respond to my suggestions. I hope your work contributes to the broader learning and teaching.

[Response]: We thank the reviewers for their comments and insights, which we believe have substantially improved the quality and clarity of our manuscript.

**Reviewer 4**

Abstract:
The abstract effectively highlights the gap in qualitative research on high-impact practices (HIPs) for second-year students, providing a compelling rationale for the phenomenographic approach. To enhance readability, consider briefly specifying one example of a 'positive outcome' in each dimension to give readers a more vivid preview of the results."

[Response]: Thank you for this suggestion. While we agree that additional examples in the abstract could be beneficial, providing such examples would cause us to exceed the word limit (300 words).

We believe that providing brief examples would also risk oversimplifying the nuanced and contextually situated nature of our qualitative findings. As such, we have designed the abstract to serve as a conceptual roadmap that invites readers to explore this complexity rather than offering a reductive and superficial snapshot. The findings section is where it is best to save the examples so we can faithfully preserve the power of student voice that is central to our study.

Elaborate slightly on how the summer-bridge program's design influenced these outcomes (e.g., activity types), as this could strengthen the link to practical recommendations without exceeding word limits."

[Response]: Thank you for this suggestion. We have revised the paragraph beginning on Line 200 to include more details on program activities and noted that students frequently reflected on these activities in their weekly reflections.

Condense the discussion of first-year comparisons (lines 88-94) slightly to allocate more space for emerging trends in second-year retention, such as post-pandemic effects, to underscore timeliness."

[Response]: Thank you for this suggestion. We have found that the second-year retention rates have been largely similar over the past decade, with slight upward trends post-pandemic. We have noted these statistics in this paragraph (beginning on Line 88) from the National Student Clearinghouse Research Center and American Institutes for Research.

For greater impact, suggest explicitly noting potential overlaps between dimensions (e.g., how social support influences personal efficacy, as hinted in lines 191-192) to invite future intersectional analyses."

[Response]: Thank you for this suggestion. We have added to the second paragraph in the limitations section, where we discuss future directions (beginning on Line 652), to further elaborate on potential overlaps between dimensions and the importance of future intersectional analyses of these dimensions to better understand high-impact practices that support holistic student development.

To extend applicability, I recommend incorporating a brief discussion of how these challenges manifest in nursing and paramedical students, who often grapple with unique educational hurdles like integrating clinical simulations, managing high-stakes ethical dilemmas, and balancing rigorous coursework with early fieldwork—potentially adapting HIPs to boost clinical confidence and reduce attrition in these high-demand fields." The following references are suggested for writing this paragraph: Afshari A, Khodaveisi M, Sadeghian E. Exploring the educational challenges in emergency medical students: A qualitative study. Journal of Advances in Medical Education & Professionalism. 2021 Apr;9(2):79.

[Response]: We appreciate this thoughtful suggestion to increase the impact of our findings. We have added a final sentence in the limitations section acknowledging that future research could examine how high-impact practices support students across different academic and disciplinary contexts, including those with specialized training requirements. We have also included the aforementioned reference. However, given the focus of our study is on second year undergraduate students and the need to maintain a reasonable manuscript length, we felt that a more detailed discussion of discipline-specific challenges in such health professions programs would be beyond the scope of this paper.

Why did you use the phenomenological method in this study? Explain.

[Response]: Thank you for this inquiry about our methodological choice. We have expanded the paragraph beginning on Line 249 to clarify our rationale for using phenomenography. We now explain that phenomenography is particularly suitable for this study because students participating in the same program activities may foreground and interpret different aspects of their experiences, and we clarify how variation theory helps us describe and catalog the range of perceived outcomes from students' engagement in high-impact practices.

For enhanced methodological contribution, suggest discussing potential biases in peer mentor checks of reflections and how they were mitigated, which could inform adaptations for health professions training where mentor feedback is common."

[Response]: Thank you for this suggestion. We have clarified in the paragraph beginning on Line 292 that peer mentor checks were administrative rather than evaluative. Peer mentors checked reflections for completion and verified that students were participation in appropriate activities. However, mentors were not part of the research team and did not provide substantive feedback that would have influenced the content of the reflections, which reduced the potential of biasing or influencing students' self-reported experiences.

Describe in detail how you selected the samples. Which of the purposive methods did you use?

[Response]: Thank you for this question. We have clarified in the paragraph beginning on Line 222 that our sampling approach combined convenience and purposive sampling. We now explicitly identify that we used criterion sampling by selecting a program where students engaged in high-impact practices across four dimensions and submitted structured reflections on these experiences.

We look forward to the review process and to providing any additional information that would be helpful. Thank you very much for your time and thoughtful consideration.

Sincerely,

Stanley M. Lo, Ph.D.

Teaching Professor, Cell and Developmental Biology

Co-Director, Joint Doctoral Program in Mathematics and Science Education

Affiliate Faculty, Research Ethics Program

University of California San Diego
